# EDTA chelation therapy for cardiovascular disease: a systematic review

**DOI:** 10.1186/1471-2261-5-32

**Published:** 2005-11-01

**Authors:** Dugald MR Seely, Ping Wu, Edward J Mills

**Affiliations:** 1 Canadian College of Naturopathic Medicine, Toronto, Canada; 2 Institute of Medical Science, University of Toronto, Toronto, Canada; 3 London School of Hygiene and Tropical medicine, University of London, UK; 4 Department of Clinical Epidemiology & Biostatistics, McMaster University, Hamilton, Canada

## Abstract

**Background:**

Numerous practitioners of both conventional and complementary and alternative medicine throughout North America and Europe claim that chelation therapy with EDTA is an effective means to both control and treat cardiovascular disease. These claims are controversial, and several randomized controlled trials have been completed dealing with this topic. To address this issue we conducted a systematic review to evaluate the best available evidence for the use of EDTA chelation therapy in the treatment of cardiovascular disease.

**Methods:**

We conducted a systematic review of 7 databases from inception to May 2005. Hand searches were conducted in review articles and in any of the trials found. Experts in the field were contacted and registries of clinical trials were searched for unpublished data. To be included in the final systematic review, the studies had to be randomized controlled clinical trials.

**Results:**

A total of seven articles were found assessing EDTA chelation for the treatment of cardiovascular disease. Two of these articles were subgroup analyses of one RCT that looked at different clinical outcomes. Of the remaining five studies, two smaller studies found a beneficial effect whereas the other three exhibited no benefit for cardiovascular disease from the use of EDTA chelation therapy. Adverse effects were rare but those of note included a few cases of hypocalcemia and a single case of increased creatinine in a patient on the EDTA intervention.

**Conclusion:**

The best available evidence does not support the therapeutic use of EDTA chelation therapy in the treatment of cardiovascular disease. Although not considered to be a highly invasive or harmful therapy, it is possible that the use of EDTA chelation therapy in lieu of proven therapy may result in causing indirect harm to the patient.

## Background

Chelation therapy, a program of repeated intravenous administration of ethylene diamine tetraacetic acid (EDTA), often given in combination with vitamins and minerals, has been touted as a safe alternative treatment for atherosclerotic vascular disease [1-3]. As this is a non-conventional therapy, there is no universally recognized standard protocol. Most protocols, however, share a degree of similarity. A typical protocol might consist of 30 intravenously administered solutions of 3 grams of disodium EDTA with concomitant administration of varying levels of ascorbic acid, B-vitamins, heparin, and the minerals magnesium, copper, zinc, selenium and manganese delivered over 1.5 to 3 hours in 500 ml to 1000 ml of normal saline. Therapy is often delivered on a weekly or biweekly basis and may be followed up with a less frequent maintenance schedule.

There is a very strong market for this therapy with out-of-pocket costs for the use of EDTA to treat cardiovascular disease estimated to range from $400 million to $3 billion annually in the United States [4,5]. The use of chelation therapy for cardiovascular disease appears to contradict conventional medical thought, however, as three systematic reviews of clinical trials have concluded that chelation therapy is not supported by the evidence [1,2,6]. In contrast, one early meta-analysis of uncontrolled trials and unpublished data claimed that EDTA chelation therapy effectively improved the symptoms of cardiovascular disease in over 80% of cases [7].

There is continued controversy as to the use of this therapy and a substantial amount of press given to it in popular non-peer reviewed literature and on the Internet. Considering the continued widespread usage and interest in EDTA chelation therapy we have endeavored to review the most current state of the evidence in order to provide an update on this contentious and clinically relevant issue.

## Methods

### Search strategy

We searched the following databases, from inception to May 2005: MedLine, EMBASE, Cochrane controlled trials register (CENTRAL), AMED (Alternative Medicine);Alt HealthWatch;Pre-CINAHL;CINAHL;and the Nursing and Allied Health Collection. In addition we searched for unpublished and on-going trials through *Clinicaltrials*. gov and the National Research Register (UK). To be included in this review, a trial had to be a randomized controlled trial assessing EDTA in humans at risk for cardiovascular disease. We excluded non-randomized trials and pharmacokinetic studies. No limits based on language were imposed. Two reviewers (DS and PW) independently assessed the articles for inclusion and outcome data. In the case of disagreement, arbitration was sought from EM.

A more detailed description of the search strategy used and the results are presented [see additional file 1]. Authors of some of the trials were contacted to solicit their interpretation of the review and also to comment on any criticism that we had found in non-peer reviewed literature.

### Data abstraction

We abstracted outcome data on ECG tests, exercise tests including treadmill tests, cycling time, time to ischemia, pain free and maximal walking distances, subjective symptoms of angina, ankle brachial indices, digital subtraction angiograms, transcutaneous oxygen tension, blood cholesterol levels, and quality of life measures. We planned to conduct a meta-analysis, however, the limited number of trials, their clinical heterogeneity, and the variability of outcomes made pooling impossible. We abstracted quality criteria data from each of the RCTs assessed based on randomization, sample size determination, dropouts, intention to treat, blinding, and allocation concealment.

## Results

### Main findings

We found a total of fourteen studies for further analysis [4,8-20]. Of these, we excluded seven, as they were either review articles or non-randomized studies [8-14]. Seven studies of randomized controlled trials have been included in this systematic review [4,15-20]. Of the seven manuscripts, five were of distinct sample populations [4,16-18,20], with three of the trials analyzing data from only one participant sample [15,16,19]. Except for one trial published in Danish, all trials were published in English between 1963 and 2002. The data from the Danish trial was also published in English, however, thereby not requiring translation or inclusion. All included trials were conducted in the United States, Canada, Denmark, New Zealand, or Germany.

Of the five sample populations tested, two studies (total n = 19) demonstrated a beneficial effect of EDTA chelation therapy on cardiovascular disease measures and three (total n = 269) did not.

### Study characteristics and adverse effects

Since the most recent systematic review, a Cochrane review, by Villaruz et al., 2002 [6], there has been one randomized controlled clinical trial on the effect of EDTA on cardiovascular disease. The conclusion found by Villaruz et al was that 'there is insufficient evidence to decide on the effectiveness or ineffectiveness of chelation therapy in improving clinical outcomes of patients with atherosclerotic cardiovascular disease' [6].

The most recent placebo-controlled trial conducted by Knudtson and colleagues in 2002 [4] explored the effect of chelation therapy on ischemic heart disease. When compared to placebo, a total of 33 rounds of EDTA treatment per patient was found to have no effect in any of the outcomes measured [see additional file 2]. Both groups exhibited improvements from baseline but this was independent of EDTA use.

Two of the earliest randomized controlled trials, by Kitchell et al., 1963 [17] and Olszewer et al., 1990 [18], found EDTA chelation therapy to have a beneficial effect on cardiovascular risk profiles when compared to control. In the first trial by Kitchell et al., 50% of the active group experienced improved ECG readings after 6 and 12 weeks following the final treatment. This crossover trial was halted early because 3 of the 5 members in the placebo group dropped out due to a lack of improvement. In the trial by Olszewer et al. (1990), the code was broken early because the exercise measures and the ankle brachial blood pressure index all showed dramatic improvements in the chelation group [see additional file 2]. Of all the trials analyzed, however, these studies used the least number of participants, with sample sizes of 9 and 10 respectively.

Since the two positive trials by Kitchell and Olszewer, three larger trials have been completed. None of the more recent trials has indicated any benefit from EDTA therapy on cardiovascular disease. One group of investigators, Guldager et al., 1992 [16], tested 153 patients with intermittent claudication for a number of clinical outcomes [see additional file 2]. None of these outcomes demonstrated evidence of improvement using parenteral EDTA chelation. Two subsets of this initial sample were analyzed separately; one for digital angiograms and transcutaneous oxygen profiles [19], and one for blood cholesterol levels [15]. In neither of these outcomes was there any evidence of improvement dependent the intervention alone. In the trial by Van Rij et al., 1994 [20], 32 patients were tested for similar outcomes with the same negative findings. As described above, the most recent trial by Knudtson et al., 2002 did not find any evidence of efficacy in the treatment of 84 patients with coronary artery disease through EDTA chelation therapy [4].

We assessed for adverse effects amongst the trials and found only a few cases of adverse events that might be attributed to the EDTA [see additional file 2]. In the EDTA treatment (vs. control) groups, one trial had a single (zero controls) case of potential kidney toxicity in a population of 84 [4] and in another trial, eight cases (two controls) of faintness and 12 cases of hypocalcemia (one control) in a population of 153 were found [16]. No other adverse effects were noted in any of the RCTs.

### Study quality

In an assessment of the articles for quality criteria, two of the trials did not describe randomization [15,17]; sample size determination was only described in two trials [4,20]; drop outs were excessive in one [17]; intention to treat analysis was performed in four trials [4,18-20]; blinding was inadequate in two of the trials [17,18]; and allocation concealment was only adequate in one trial [4]. A summary of quality criteria findings is listed in table 1. The two trials with the highest quality [4,20] were the most recent and, as discussed above, found no evidence of efficacy for EDTA chelation therapy in the treatment of cardiovascular disease.

**Table 1:** Quality criteria of the randomized controlled trials assessed

**Reference**	**Randomization**	**Sample size determination**	**Drop outs**	**Intention to treat analysis**	**Blinding**	**Allocation concealment**
Kitchell 1963 [17]	Not described (nor stated)	Not described	33% of the treatment group upon cross-over	No	Allegedly participants blinded, but impossible to adequately assess	Unclear
Olszewer 1990 [18]	Described	Not described	None	Yes	Initially double blinded but code was broken after 10 treatments and study was completed with single blind for remaining 10 treatments	Unclear
Sloth-Nielsen 1991 [19]	Described	Not described	None	Yes	Double	Unclear
Guldager 1992 [16]	Described	Not described	4 drop outs by 3 months; 30 drop outs by 6 months	No	Double: code broken at 3 months	Unclear
Guldager 1993 [15]	Not described	Not described	Not mentioned	No	Double	Unclear
Van Rij 1994 [20]	Described	Yes	None	Yes	Double	Unclear
Knudtson 2002 [4]	Described	Yes	4 in placebo, 2 in treatment group	Yes	Double	Well described

## Discussion

The overall evidence on EDTA chelation therapy argues against any clinical benefit with respect to cardiovascular disease. The evidence that we were able to find in support of EDTA chelation for cardiovascular disease relies almost entirely on uncontrolled trials and a large body of anecdotal evidence. Given the parameters of the evidence so far, and in keeping with patient values, it is encouraging that the NIH is funding a large simple trial. A 5-year multi-center randomized placebo-controlled trial will hopefully deliver a conclusive answer on the question of whether or not intravenous EDTA infusion has any role in the treatment of cardiovascular disease. Until such time, clinicians should openly discuss the use of complementary and alternative medicine and specifically chelation therapy with patients in an informed and non-judgmental manner. Clinicians should also discuss potential risks associated with EDTA chelation therapy and the current lack of evidence supporting it use in cardiovascular disease.

Proposed mechanisms of action for the reversal of cardiovascular disease by EDTA include: calcium chelation resulting in dissolution of atheromatous plaques, free radical scavenging action, reduction of total body iron stores, cell membrane stabilization, arterial dilatation due to calcium channel blocking action, improvement of arterial wall elasticity and increased production of nitric oxide [4,21]. Critics have taken issue with some of these mechanisms, however, claiming the use of outdated concepts on the pathophysiology of atherosclerosis and for instance, the inability of EDTA, a water soluble compound, to effectively complex with plaque calcium [3].

Given the widespread usage of EDTA chelation therapy, an assessment of its safety is crucial. EDTA is responsible for a wide range of potential side effects including gastrointestinal and musculoskeletal complaints, diaphoresis, fever, leukopenia, thrombocytopenia, kidney damage, mineral depletion, and hypocalcemia [19,22-24]. With proper dose control and assessment of kidney function, however, EDTA is not considered to be a particularly high-risk therapy and there is little doubt that it is safer than coronary bypass surgery. However, if EDTA has no efficacy beyond placebo, then the possibility of adverse effects, the cost of treatment, and potential to avoid greater risk may well result in unjustifiable morbidity and mortality.

There are several limitations to consider in our review. We cannot determine to what extent publication bias has affected our results. Empirical evidence has shown that negative trials in CAM and other fields are less likely to be published [25]. Claims have been made however, that evidence in favor of EDTA chelation therapy may have been suppressed [26]. To mitigate as much as possible against these circumstances, we conducted thorough systematic searches of the literature. We contacted some, but not all of the authors of the studies to confirm our interpretation of the results. It may be that authors of original studies excluded important information from the published manuscripts in order to reduce the page length or to follow reviewers' suggestions [27]. In many of the trials there was a significant improvement in outcome measures for both control groups and chelation groups. This is a clear indication of the need to conduct controlled trials in order to pick up a type I error that might mistakenly attribute efficacy to the therapy in question.

There is a large body of literature to support the use of EDTA in the treatment of cardiovascular disease; however, the vast majority of the literature relies on uncontrolled evidence. Supporters of EDTA chelation therapy have levied a number of criticisms against some of the RCTs assessed in this review. These criticisms include but are not limited to: too short of a treatment schedule, claims of incorrect statistical manipulation of the data, improper randomization, and high dropout rates [26,28-30]. The earliest trials incorporated 20 treatments of chelation therapy and the American College for Advancement in Medicine (ACAM), considered an authority in chelation therapy, claims that at least 30 treatments may be required for improvements to be noted. In the most recent study by Knudston et al., 2002, however, a protocol involving 33 treatment sessions were used and no positive therapeutic effect was found. In our review we included quality criteria and did not find evidence of any bias, however, we did not obtain the raw data needed to conduct an independent reanalysis.

## Conclusion

The findings of this systematic review should be of interest to clinicians and patients alike. The use of EDTA by patients as a treatment for cardiovascular disease and as an adjunct or alternative to surgery is not supported by the highest quality of evidence. Considering the cost incurred by patients who use EDTA chelation therapy and the potential for harm associated with any intravenous intervention including the potential for adverse effects attributable directly to EDTA, clinicians should inquire about patient use and highlight the lack of evidence to support its usage.

## List of abbreviations used

CAM: complementary and alternative medicine

ECG: electrocardiogram

EDTA: ethylene diamine tetraacetic acid

NIH: National Institutes of Health

RCT: randomized controlled trial

## Competing interests

The author(s) declare that they have no competing interests.

## Authors' contributions

DS and EM conceived the design of the study, DS and PW conducted the searches and undertook the data extraction and analysis with assistance from EM. DS drafted the manuscript. EM edited the manuscript.

**Figure 1:**
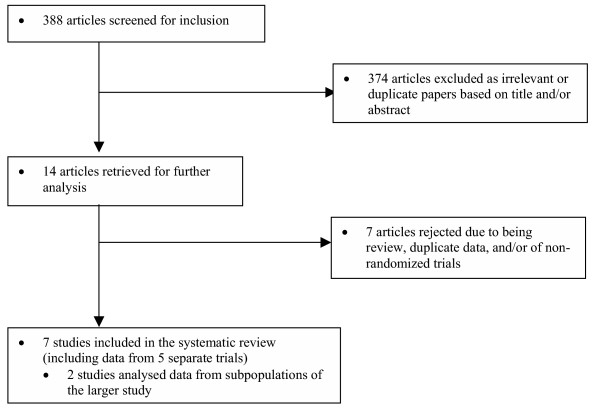
Flow-chart detailing study selection and exclusion for systematic review.

## Pre-publication history

The pre-publication history for this paper can be accessed here:



## Supplementary Material

Additional File 1 (text) EDTA and cardiovascular disease systematic review search strategy employed. Click here for file

Additional File 2 (table) Randomized controlled trials assessing the therapeutic use of EDTA for atherosclerotic cardiovascular disease. Click here for file
