# Supplementary material for: EDTA chelation therapy for cardiovascular disease: a systematic review
Source: BMC Cardiovasc Disord. 2005 Nov 1;5:32. doi: 10.1186/1471-2261-5-32 (PMC1282574; doi:10.1186/1471-2261-5-32)
Supplement: Additional File 1 — (text) EDTA and cardiovascular disease systematic review search strategy employed. [file 1471-2261-5-32-S1.doc]

# EDTA and cardiovascular disease systematic review

# Search strategy employed

# July 4, 2005: Medline

1. EDTA or chelation or “ethylene diamine tetraacetic acid”
2. cardiovasc* or corona* or atherosc* or circulat* or heart or CAD
3. peripher* or intermit* or claud* or PAD
4. #1 and (#2 or #3)
5. RCT or random* or control* or trial or clinical
6. #4 and #5 🡪 949 citations found
7. CAM or complement* or alternat*
8. **#6 and #7 🡪 68 citations assessed (4 full articles tagged for retrieval)**
9. **#6 (limits set to randomized controlled trials) 🡪 67 citations assessed (8 full articles tagged for retrieval)**
10. **#6 and (RCT or randomized controlled trial) 🡪 73 citations assessed (10 full articles tagged for retrieval)**

**July 4, 2005:** AMED (Alternative Medicine); Alt HealthWatch;  Pre-CINAHL;  CINAHL;  Nursing and Allied Health Collection

1. EDTA or chelation or “ethylene diamine tetraacetic acid”
2. cardiovasc* or corona* or atherosc* or circulat* or heart or CAD
3. peripher* or intermit* or claud* or PAD
4. #1 and (#2 or #3)
5. RCT or random* or control* or trial or clinical
6. **#4 and #5 🡪 19 citations assessed (2 full articles tagged for retrieval)**

# July 4, 2005: EMBASE

1. EDTA or chelation or “ethylene diamine tetraacetic acid”
2. cardiovasc$ or corona$ or atherosc$ or circulat$ or heart or CAD
3. peripher$ or intermit$ or claud$ or PAD
4. #1 and (#2 or #3)
5. RCT or random$ or control$ or trial or clinical
6. **#4 and #5 🡪 87 citations assessed (5 full articles tagged for retrieval)**

# July 4, 2005: CENTRAL

1. EDTA or chelation or “ethylene diamine tetraacetic acid”
2. cardiovasc* or corona* or atherosc* or circulat* or heart or CAD
3. peripher* or intermit* or claud* or PAD
4. **#1 and (#2 or #3) 🡪 74 citations assessed (10 full articles retrieved)**

Of 39 articles tagged for retrieval, 14 were unique. Of these, 7 were excluded and 7 were included.

- Reasons for exclusion:
- Review article
- Not randomized
- Duplicate data

No language restrictions were imposed for any of the searches
