# Supplementary material for: EDTA chelation therapy for cardiovascular disease: a systematic review
Source: BMC Cardiovasc Disord. 2005 Nov 1;5:32. doi: 10.1186/1471-2261-5-32 (PMC1282574; doi:10.1186/1471-2261-5-32)
Supplement: Additional File 2 — (table) Randomized controlled trials assessing the therapeutic use of EDTA for atherosclerotic cardiovascular disease. [file 1471-2261-5-32-S2.doc]

Additional file 2: All randomized controlled trials assessing the therapeutic use of EDTA for atherosclerotic cardiovascular disease.

| **Reference** | **Participants** | Intervention | **Control** | **Outcomes measured** | **Results** | **Adverse Effects** |
| --- | --- | --- | --- | --- | --- | --- |
| **Kitchell 1963[17]** | 9 patients with severe angina due to coronary artery disease (subset of a larger uncontrolled group) | 20 treatments of EDTA (3-4 g) in 500 ml isotonic glucose or saline solution 3-4x /wk for 6-8 wks | Placebo undefined | 1. Treadmill exercise test 2. ECG evaluation 3. Subjective symptoms of angina | Treatment: Claims of improvement in angina symptoms 6 and 12 wks post final infusion. Improved S-T segment and T wave changes in 2 of 4 in active group.  Control: No improvements seen | Not mentioned |
| **Olszewer 1990[18]** | 10 males with intermittent claudication | 20 treatments of EDTA 10 ml (1.5 g), ascorbic acid (2 g), B-complex (2 ml), Vit B6 (300 mg), heparin (500 IU), and MgSO_4_. Code broken after 10 treatments | 10 ml distilled water, ascorbic acid (2 g), B-complex (2 ml), Vit B6 (300 mg), heparin (500 IU), and MgSO_4_ | 1. Exercise parameters including walking time, number of steps, and cycling time 2. Ankle brachial index | Statistically significant improvement over placebo with EDTA treatment after 10 treatments in all exercise measures (mean improvement factor = 2.1) and in the ankle brachial index (mean improvement factor = 1.4 at rest and after exercise) | Not mentioned |
| **Sloth-Nielsen 1991[19]** | 30 patients with intermittent claudication (subset of the Guldager 1992[16] study) | 20 treatments of EDTA (3 g) in 1L isotonic saline solution over 6-10 wks | 1 L isotonic saline solution | 1. Digital subtraction angiograms 2. Transcutaneous oxygen tension measurements | No differences in all measures between treatment and placebo groups | Non specific and no difference between groups |
| **Guldager 1992[16]** | 153 patients with intermittent claudication | 20 treatments of EDTA (3 g) in isotonic saline with daily oral multivitamins and minerals for 5-9 wks | 1 L isotonic saline solution with daily oral multivitamins and minerals | 1. Subjective evaluation 2. Pain-free and maximal walking distances 3. Ankle-brachial pressure index | No differences in all measures between treatment and placebo groups | 6 cases of hypocalcemia and 11 cases of faintness in active group versus 2 cases and 1 case in placebo respectively |
| **Guldager 1993[15]** | 29 patients with Intermittent claudication (subset of the Guldager 1992[16] study) | 20 treatments of EDTA (3 g) in 1L isotonic saline solution with daily oral multivitamins and minerals | 1L isotonic saline solution with daily oral multivitamins and minerals | 1. Plasma concentrations of total cholesterol, HDL, LDL and triglycerides | No differences in all measures between treatment and placebo groups | Not mentioned |
| **Van Rij 1994[20]** | 32 patients with intermittent claudication | 20 treatments of EDTA (3 g), MgCl (0.76 g), NaHCO_3_ (0.84 g), and B vitamin complex in 500ml normal saline 2x/wk for 10 wks with daily oral multivitamin | 500 ml normal saline with B-vitamin complex with daily oral multivitamin | 1. Walking distance 2. Subjective evaluation 3. Ankle/brachial indices | No differences in all measures between treatment and placebo groups | None found |
